# Unraveling the Antibiofilm Activity of a New Nanogold Resin for Dentures and Epithesis

**DOI:** 10.3390/pharmaceutics14071513

**Published:** 2022-07-21

**Authors:** Vera Ivanovic, Danica Popovic, Sanja Petrovic, Rebeka Rudolf, Peter Majerič, Milos Lazarevic, Igor Djordjevic, Vojkan Lazic, Milena Radunovic

**Affiliations:** 1 Laboratory for Microbiology, School of Dental Medicine, University of Belgrade, 11000 Belgrade, Serbia; vera.ivanovic@yahoo.com; 2 Clinic for Prosthodontics, School of Dental Medicine, University of Belgrade, 11000 Belgrade, Serbia; d.popovic984@gmail.com (D.P.); igor.djordjevic@stomf.bg.ac.rs (I.D.); vojkan.lazic@stomf.bg.ac.rs (V.L.); 3 Faculty of Mechanical Engineering, University of Maribor, 2000 Maribor, Slovenia; rebeka.rudolf@um.si (R.R.); peter.majeric@um.si (P.M.); 4 Department of Human Genetics, School of Dental Medicine, University of Belgrade, 11000 Belgrade, Serbia; milos.lazarevic@stomf.bg.ac.rs

**Keywords:** PMMA, gold nanoparticles (AuNPs), biofilm, antibiofilm effect

## Abstract

Dentures and epitheses are mostly made from poly(methyl methacrylate) (PMMA), which does not show antimicrobial properties. They present reservoirs of microorganisms grown in biofilms. The aim of this study is to prepare a PMMA enriched with gold nanoparticles (AuNPs)-PMMA/AuNPs and the examination of its physical, mechanical and antimicrobial properties. The AuNPS were synthetized from HAuCl_4_ using the ultrasonic spray pyrolysis method with lyophilization. The PMMA/AuNP samples were compared to PMMA samples. Density was measured by pycnometer. Microhardness was evaluated using the Vickers hardness test. Monomicrobial biofilm formation (*Streptococcus mitis*, *Candida albicans*, *Staphylococcus aureus* and *Escherichia coli*) was measured by colony-forming units (CFUs) and MTT test and visualized by SEM. AuNP release was measured indirectly (the CFUs of the medium around the sample). The density and microhardness of the PMMA/AuNPs were similar to those of the PMMA. CFU and MTT values for the biofilms formed on the PMMA for each of the tested species were higher than those of the biofilms formed on the PMMA/AuNPs. The CFUs of the medium around the sample were similar for both materials. PMMA/AuNPs showed a significant reduction in the monomicrobial biofilms of all tested species. AuNPs are not released from PMMA/AuNPs. Density, indirect measurement of residual monomer and dentures weight were similar between PMMA and PMMA/AuNPs. Microhardness, as a measure of the wear resistance, was also similar between tested discs.

## 1. Introduction

The oral cavity represents one of the most diverse microbiomes of the healthy human body [1]. Within the healthy oral cavity, there are several special ecosystem niche levels, consisting of shedding epithelium and non-shedding hard tissues and, the most specific, the junction of these two tissues, the periodontal sulcus [2,3]. The oral microbiome may be disturbed by a number of diseases (i.e., diabetes), habits (i.e., food, alcohol consumption, cigarette smoking and oral hygiene) or physiological states (i.e., puberty and pregnancy) [4,5,6]. The addition of dentures also leads to changes in the oral microbiome [7], but it is usually neglected as a factor that may affect the microbiome. Dentures, artificial non-shedding surfaces in the oral cavity, are most commonly made of artificial resins, such as poly(methyl-methacrylate) (PMMA) [8]. This material is also used for epithesis/obturator prothesis [9], a prothesis used for maxillary defect reconstruction usually after neoplasm resection. Despite its good performance, poor surface properties remain a problem of PMMA [10]. Surface properties (hydrophobicity and charge), pores, cracks and structural defects as a consequence of gas release during the polymerization process [11,12] enable microorganisms to adhere to the surface, grow, form biofilms and penetrate into the denture and subsist inside the denture/obturator [8,13]. Changes in denture surface as a consequence of poor wear resistance (microhardness) are inevitable and also contribute to microbial attachment. It has been previously proven that PMMA dentures are reservoirs of microorganisms embedded in biofilms, which may disseminate to the respiratory system or even the blood stream [14]. Having this in mind and the fact that users of PMMA dentures/obturators are usually older or immunocompromised patients, it is clear that the antimicrobial properties of this material should be improved.

In order to improve the mechanism of and develop the antimicrobial characteristics of PMMA, there have been attempts to modify it. These modifications include the addition of fiber materials (i.e., glass fibers and polyethylene fibers) and fillers (e.g., aluminum-oxide, zirconium-oxide, silver and hydroxyapatite) [15]. It has been proven that silanized glass fiber improves flexural strength and biocompatibility [16,17], while decreasing porosity and the adherence of *Candida albicans* [18]. PMMA with incorporated zinc oxide nanoparticles has been shown to be biocompatible and has improved fractural strength [19]. Furthermore, silver nanoparticles incorporated into PMMA increase the compressive strength, viscoelastic strength and thermal conductivity of PMMA [20,21]. The results of the antimicrobial effect of PMMA enriched with nano-silver are conflicting. Some studies showed a reduced adhesion and growth of *C. albicans* [22], while others failed to show this effect [23]. The antibacterial effect of silver nanoparticles against *Staphylococcus aureus*, *Pseudomonas aeruginosa* and *Escherichia coli* has been proven for other materials, such as food packaging and hemostatic sponges [24,25]. Even though all of the above-mentioned PMMA modifications have shown some improvement in their characteristics, none have met all the requirements for an ideal material for dentures/epithesis, especially regarding antimicrobial characteristics.

Among various metallic nanoparticles, there has been an increased interest in gold nanoparticles (AuNPs) due to their favorable characteristics. These characteristics include low toxicity, small size, precise targeting and relatively simple fabrication [26]. Another very favorable quality that has recently attracted the interest of researchers is the antibacterial properties of AuNPs. The antimicrobial effects of AuNPs have been demonstrated through various mechanisms. Smaller AuNPs produce holes in the bacterial cell wall, which leads to the loss of cell contents and eventually bacterial cell death [27,28]. Larger AuNPs (in a range of 80–100 nm in size) adhere on bacterial cells, causing an increase tension in the membrane, the deformation of the cell and eventually the cell’s rupture [29]. Additionally, AuNPs bind to bacterial DNA and stop transcription by blocking the uncoiling of bacterial DNA [27]. Some authors describe that AuNPs may hinder ATPase activity, causing the deterioration of cell metabolism or may hinder the binding subunit of the ribosome to the tRNA [30,31]. In addition, AuNPs may affect the bacterial respiratory chain through nicotine amide [32] or generate reactive oxygen species and induce oxidative stress [31,33]. A recent study showed that AuNPs have a strong antibacterial effect on Gram-negative bacteria and a moderate activity against Gram-positive bacteria [34]. In addition to having an antimicrobial effect in solutions, the ability of AuNPs to reduce biofilm formation on surfaces has also been studied recently. Ali et al. demonstrated that the ability of *Pseudomonas aeruginosa* to form biofilms was greatly affected by AuNPs by demonstrating that, with increasing the concentration of AuNPs, there was a decrease in the number of biofilm-forming bacterial cells [35]. Additionally, cement materials with incorporated AuNPs showed antibiofilm properties against methicillin-resistant *Staphylococcus aureus* (MRSA) and *Pseudomonas aeruginosa* [12].

Having this in mind, we combined PMMA with AuNPs to create a new material that would have a preventive effect on biofilm formation, protecting oral and facial tissues from pathogenic yeasts and bacteria. The aim of this study is to compare the antibiofilm properties of PMMA enriched with AuNPs to conventional PMMA, as well as their density and microhardness.

## 2. Materials and Methods

### 2.1. Material Areparation

Two materials were tested in this study: poly(methyl-methacrylate) (PMMA) (PMMA-Biogal^®^, Galenika, Belgrade, Serbia) and the newly developed PMMA enriched with AuNPs (delivered by Zlatarna Celje d.o.o. Celje, Slovenia). AuNPs were in the dried form, with a chloride precursor being used as starting material for ultrasonic spray pyrolysis (USP) and lyophilization for drying. Polyvinylpyrrolidone (PVP) was added as a stabilizer into the USP-collecting medium, deionized water, in a concentration of 5 g/L. The final purity of AuNPs was high 99.99 wt%.

#### 2.1.1. Preparation of PMMA Materials

Poly(methyl-methacrylate) (PMMA) (PMMA-Biogal^®^, Galenika, Belgrade, Serbia) was prepared as recommended by the manufacturer (23.4 g of powder with 15 mL of liquid monomer).

#### 2.1.2. Development of the PMMA/AuNP Composite Material

The PMMA/AuNP composite material was developed by incorporating liquid AuNPs in the commercially used PMMA (PMMA-Biogal^®^, Galenika, Belgrade, Serbia). The AuNPs were produced from a HauCl_4_ precursor, using the ultrasonic spray pyrolysis method. They were stabilized in PolyVinilPyrolidone (PVP) and dried with the lyophilization process [36]. The dried AuNPs (>99.99%, dark brown color) were dissolved in ethanol as medium in a concentration of 1 g/L. A total volume of 15 mL of liquid (10 mL of PMMA monomer and 5 mL of liquid AuNPs) was mixed with 23.4 g of PMMA powder. The solution was stabilized with PVP. The preparation of both materials was as recommended by the manufacturer: Powder and liquid were mixed until a homogenous dough was ready for packing into cuvettes with previously prepared molds. The cuvettes were closed (80 bars pressure) and placed in water, which was gradually heated to 100 °C. After keeping it on this temperature for 1 h and 45 min, cooling to room temperature was gradual.

### 2.2. Specimen Preparation

Both materials were prepared as following:

1. Lamina dimensions 80 × 10 × 4 mm (for microhardness and density measurement):
  i. A total of 12 were made from PMMA;
  ii. A total of 12 were made from PMMA/AuNPs.
2. A total of 64 discs diameters 5 mm and thickness 2 mm for antibiofilm evaluation:
  i. A total of 32 control discs were made from PMMA;
  ii. A total of 32 test discs were made from PMMA/AuNPs.

The discs were cut by epilog Helix 40 watt (Golden, CO, USA), from the previously prepared 2 mm thick lamina dimension of 5 × 5 cm. The discs were exposed to UV light for 30 min on each side.

### 2.3. Microhardness Measurements

Surface hardness was determined using a Vickers microhardness tester (ZwickRoell ZHV10 hardness tester, Kennesaw, GA, USA), which was adjusted to a load of 0.49 N for 5 s indentation time.

### 2.4. Density Measurements

Density measurements were performed using a pycnometer by the standard procedure with the standard equation. Measurements were carried out at a temperature of 20 °C. For the density calculations, the average values of the sample mass (PMMA pure, PMMA/AuNPs 2%) and average value mass of a pycnometer with liquid (H_2_O density = 1 g/cm^3^, Ethanol density = 0.79 g/cm^3^) were calculated.

### 2.5. SEM/EDX Investigations

The AuNP and PMMA/AuNP samples were examined with SEM microscopes Quanta 200 3D (FEI, Hillsboro, OR, USA) and Sirion 400NC (FEI, Hillsboro, OR, USA) with an Energy-Dispersive X-ray spectroscope INCA 350 (Oxford Instruments, Abingdon, UK). The samples were put on SEM holders with conductive carbon adhesive tape for the examinations.

### 2.6. Antibiofilm Activity

The antibiofilm characteristics were analyzed in vitro using a total of 64 discs: 32 control discs (made from PMMA) and 32 test discs (made from PMMA/AuNPs). After biofilm formation, the antibiofilm activity was analyzed by counting colony-forming units (CFUs) and the MTT (3-(4,5-dimethylthiazol-2-yl)-2,5-diphenyl tetrazolium bromide) assay on discs. Additionally, the CFUs of the solutions around the disks after incubation were counted for all microorganisms. The last analysis was performed as an indirect indicator of the AuNP release from the discs into the solutions.

#### 2.6.1. Bacterial/Fungal Strains and Growth Conditions

The reference strains of *Staphylococcus aureus* ATCC 25923, *Escherichia coli* ATCC 25922, *Candida albicans* ATCC 10231 and *Streptococcus mitis* ATCC 6249 (Microbiologics KWIK-STIK, Manassas, VA, USA) were used. The growth conditions are presented in Table 1.

After the activation of reference strains, 3–4 colonies of *S. aureus*, *S. mitis* and *E. coli* were transferred to a brain–heart infusion (BHI) broth (HIMEDIA, India), while the same number of *C. albicans* colonies were transferred to a Sabouraud broth (HIMEDIA, India) and incubated for 24 h, on the same growth conditions. The bacterial/fungal suspension was centrifuged (10 min, 3000 rpm), the supernatant was discarded, and the pellet was resuspended in PBS (turbidity of 0.5 McFarland standard, ≈10^8^ cells/mL for bacteria and ≈10^6^ cells/mL for *C. albicans*) (DEN-1 densitometer, Biosan, Riga, Latvia). The suspensions were diluted with an enriched BHI broth (*S. mitis*, *S. aureus* and *E. coli*) or RPMI 1640 with 2% glucose medium (Sigma Aldrich, St. Louis, MO, USA) for *C. albicans*, adjusting the CFU/mL value around 10^6^ for bacteria and 105 for *C. albicans*.

#### 2.6.2. Biofilm Formation

Monomicrobial biofilms of each bacterial/fungal species were formed onto the discs. Each bacterial/fungal strain were formed on 12 discs (6 control PMMA and 6 test PMMA/AuNP discs). The control and test discs were placed in 96-well microtiter plates and kept in 100 μL of artificial saliva (Pharmacy Belgrade, Belgrade, Serbia) for 24 h at 37 °C to form a primary pellicle. After incubation with artificial saliva, monomicrobial biofilms were formed by adding 200 μL of each of the four standardized bacterial/fungal suspensions (as described in Section 2.6.1) and incubated statically at 37 °C. *S. mitis* was incubated in anaerobic conditions for 48 h, while *E. coli*, *S. aureus* and *C. albicans* were incubated in aerobic conditions for 24 h. In a separate well, 200 µL of the microorganism solution was added as a positive control, while 200 µL of broth without microorganisms served as a negative control.

##### Determination of Colony-Forming Units (CFU) of Biofilms Formed on Discs

The capacity of bacteria/fungi to form biofilms was firstly measured by counting each species’ CFUs on both material samples. The discs were washed in sterile PBS and placed in sterile plastic tubes containing 1 mL sterile PBS. Each tube was treated in an ultrasonic bath (40 kHz for 1 min) (Baku, China), followed by shaking for 10 min on vortex (900 rpm, 37 °C). Serial tenfold dilutions were seeded on Columbia agar with 5% sheep blood (*S. mitis* and *S. aureus*), Endo agar (*E. coli*) or Sabouraud agar (*C. albicans*). All the plates were incubated on 37 °C and evaluated after 24 h. *S. mitis* was incubated anaerobically, while the rest of the plates were incubated under aerobic conditions.

##### MTT Assay on Discs

The MTT assay is used to measure viable bacterial cells on biofilms formed on discs. Three discs of each material were used per each monomicrobial biofilm. After biofilm formation, the liquid around the discs was gently removed and 100 μL of MTT solution (Sigma-Aldrich) was added to each well. The plates were incubated in the dark at 37 °C for 3.5 h in statical aerobic conditions. Then, the same amount of (100 μL) of dimethyl sulfoxide (DMSO) was added and plate was shaken (250 rpm, 37 °C) for 15 min in the dark. The viability of cells was determined by measuring the colored product reflected in optical density (540 nm) on the spectrophotometer.

#### 2.6.3. Determination of Colony-Forming Units (CFUs) from the Liquid around the Discs

In order to indirectly measure the AuNP release and its antimicrobial effect in the medium surrounding the discs, we counted the CFUs in the medium. After incubation and biofilm formation on discs, ten-fold dilutions of liquid around the discs were also seeded on the solid medium (as previously described in Section Determination of Colony-Forming Units (CFU) of Biofilms Formed on Discs).

### 2.7. Scanning Electron Microscopy (SEM) for Biofilm Visualization

Scanning electron microscopy was used to visualize the monomicrobial biofilm formation of all four tested species (*S. mitis*, *E. coli*, *S. aureus* and *C. albicans*). All SEM analysis were performed in duplicate.

After biofilm formation, the discs were removed from the medium and gently rinsed in sterile PBS to remove all the unattached cells. Samples were fixed in 2.5 wt% glutaraldehyde for 24 h, followed by dehydration through a series of ascending concentrations of ethanol (0%, 25% and 50%) in 3% acetic acid. The samples were kept in 100% ethanol upon imaging.

The samples were air-dried and gold sputtered for 2 min in a JFC 1100 ion sputter (Tokyo, Japan) and then subjected to SEM imaging (JEOL JSM-840A, Tokyo, Japan), at an acceleration voltage of 20 kV.

### 2.8. Statistical Analysis

SPSS 26.0 software package for Windows (SPSS Inc., Chicago, IL, USA) was used for statistical analysis. Data distribution was tested using the Kolmogorov–Smirnov test. Differences in the CFU number and MTT analysis between biofilms on the different types of discs were analyzed using an independent sample *t*-test. *p*-value < 0.05 was considered significant.

The microhardness and density mean, standard deviation, and minimum and maximum values were calculated from a set of 12 measurements for each material property.

## 3. Results

### 3.1. Microhardness Value

The results of microhardness are presented in Table 2. Microhardness by Vickers is higher for PMMA/AuNPs, but without statistical significance.

### 3.2. Density

The results of the density measurements are presented in Table 3. There is no difference in the density of PPMA and PMMA/AuNP laminae.

### 3.3. SEM/EDX Investigations

**Figure 1:**
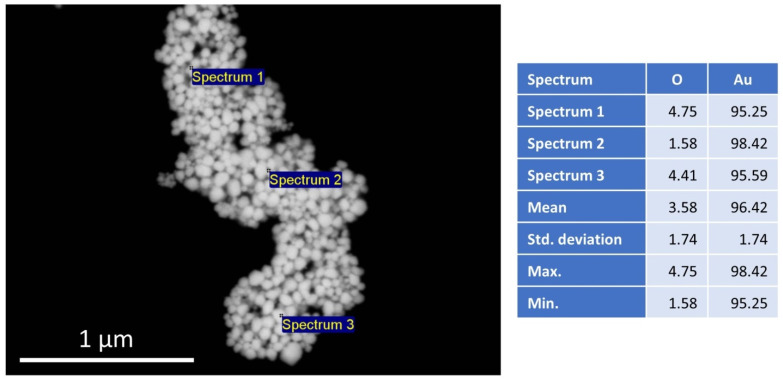
SEM image of AuNPs, with the marked locations for analysis and the resulting EDX spectrum with values in wt%.

A representative image of the SEM/EDX investigation is shown in Figure 1. The figure shows a typical morphology of the AuNPs used for the preparation of the PMMA/AuNP composite, with an EDX analysis of the elemental constituents of the particles. Several EDX point analyses show a high purity of Au, with a mean value of 96.42 wt%. The presence of O is attributed to bonds with the stabilizer, as the AuNPs were prepared by USP with PVP as the stabilizing agent. No other elements were detected.

The AuNPs were produced with the same parameters as those from a previous investigation. The mean AuNP size was 69.4 ± 12.42 nm [37].

**Figure 2:**
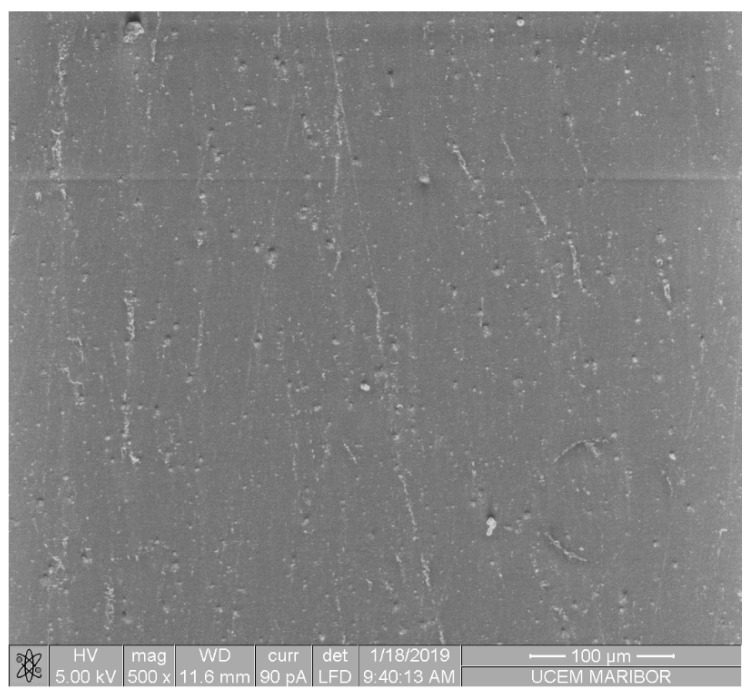
SEM image of the PMMA/AuNP composite.

Figure 2 shows the microstructure of the PMMA/AuNP composite, where it can be seen that the AuNPs are homogeneously distributed in the PMMA matrix.

### 3.4. Microbiological Assessment of PMMA and PMMA/AuNPs

**Figure 3:**
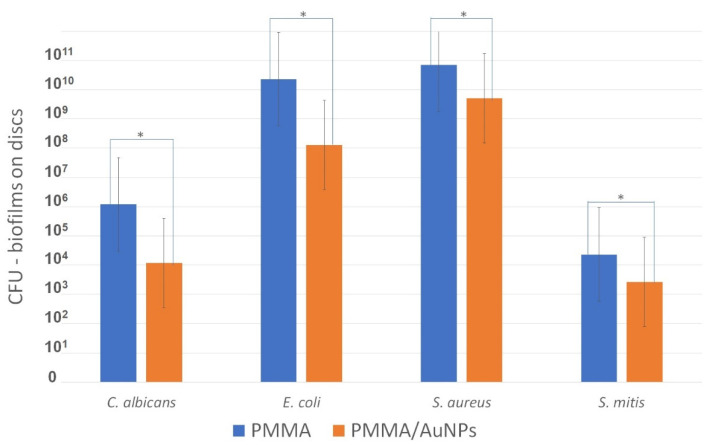
Colony-forming units (CFU) of the 24 h monomicrobial (bacterial and fungal) biofilms formed on the tested discs. * Statistically significant difference at *p* < 0.05.

The results of monomicrobial biofilm formed on the test and control discs are presented in Figure 3. The CFUs formed on the control PMMA discs for each tested species were higher than the CFUs formed on the PMMA/AuNP discs.

**Figure 4:**
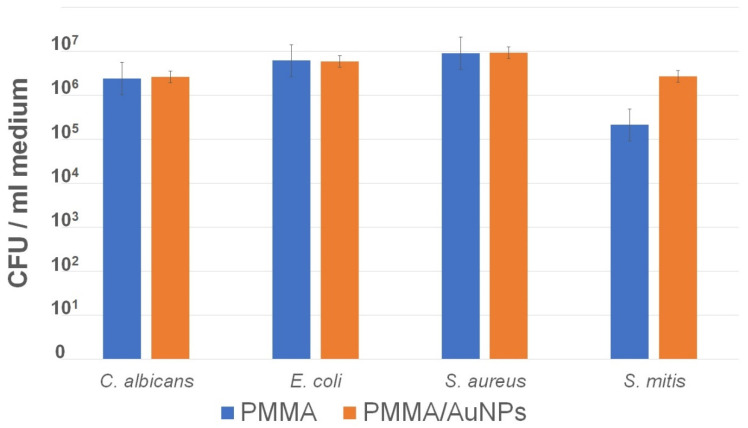
Colony-forming units (CFU) in the medium around the discs after the 24 h monomicrobial (bacterial and fungal) biofilm formation.

The CFUs measured in the medium around the test and control discs did not show statistically significant differences for any of the tested species (Figure 4).

**Figure 5:**
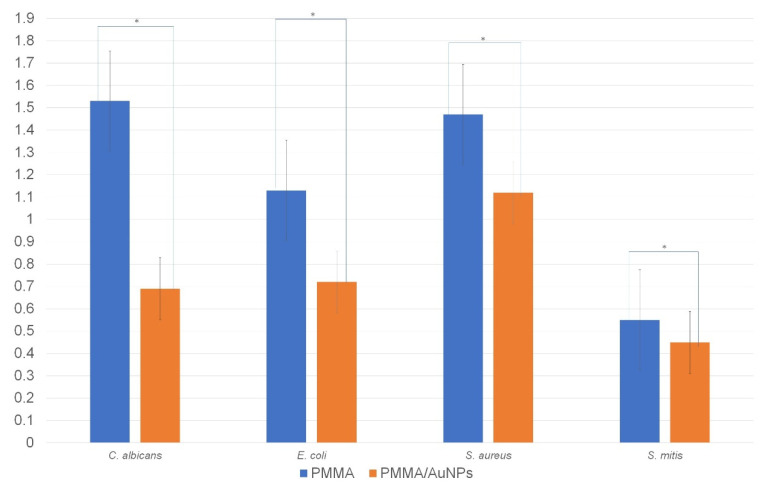
MTT results of the viability of microorganisms measured on the PMMA and PMMA/AuNP discs for all the tested species. * Statistically significant difference at *p* < 0.05.

The results of the measurement of the viability of bacteria performed by the MTT test are presented in Figure 5. Similar to the CFU results, there were statistical differences in the MTT values measured on the control and test discs.

### 3.5. Scanning Electron Microscopy Analysis for Biofilm Visualization

**Figure 6:**
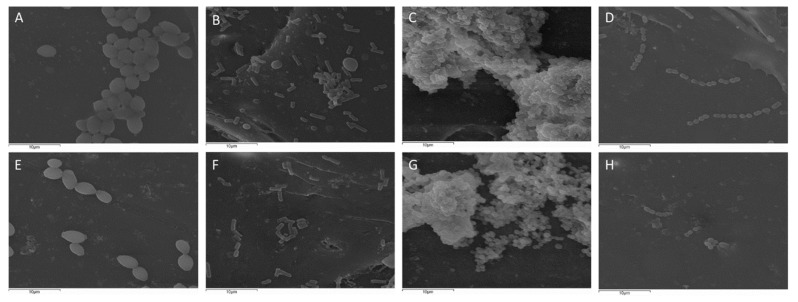
Representative scanning electron micrographs showing different microbes—(**A**,**E**) *C. albicans*, (**B**,**F**) *E. coli*, (**C**,**G**) *S. aureus* and (**D**,**H**) *S. mitis* on (**A**–**D**) PMMA and (**E**–**H**) PMMA/AuNP surfaces. Scale bars represent 10 μm, magnification 3000×.

SEM analysis showed that the number of microorganisms was lower on the PMMA/AuNP surfaces compared to those on the PMMA surfaces, similar to the results from the CFU and MTT assays. All the tested species on PMMA tend to form bigger conglomerates, while on PMMA/AuNPs, the microorganisms are dispersed individually or form pairs or small chains on the surface. Only *S. aureus* shows a biofilm structure on both materials. However, *S. aureus* biofilms formed on PMMA are larger than the biofilms formed on the PMMA/AuNPs (Figure 6).

## 4. Discussion

This study was performed to improve dental materials and provide new solutions that can be used for carriers of PMMA dentures (complete or partial) and, what is more important, obturator prothesis. The idea is to prevent or decrease the occurrence of the most frequent complication of denture/obturator wearers, denture stomatitis, as well as to lower the quantity of biofilms on these devices, which act as reservoirs of biofilms. The development of material that has the same or improved physical and mechanical characteristics as conventional PMMA and, at the same time, shows an antibiofilm effect in comparison to PMMA could decrease the frequency of denture stomatitis. Given that denture stomatitis is mostly caused by the yeast of Candida genus [38], this could significantly decrease the use of antimycotics. The reduction in antimycotic usage is important due to the following: (1) their number is limited, (2) they cause common drug interactions and show toxic effects [39], and (3) there is emerging antifungal resistance [40]. In addition to *Candida* spp., dentures’ biofilms are commonly of mixed bacterial and fungal origin, so sometimes both antibacterial and antifungal drugs are needed for treatment. At the same time, microorganisms are more resistant in mixed biofilms [41].

Most metallic nanoparticles are produced in suspensions and are chemically and physically unstable. Lyophilization as a drying technique represents a way to achieve dry nanoparticles and, with them, long-term stability at room temperature or the possibility of their use for various biomedical applications [42]. In the present study, dry AuNPs were used, which were synthesized by the USP method [43] and lyophilization for mixing into the PMMA matrix. The addition of AuNPs was also prepared with caution so as not to change the color of the PMMA, as we previously reported [44]. In this study, a decrease in biofilm formation on PMMA/AuNPs compared to conventional PMMA discs was demonstrated for the monomicrobial biofilms of *C. albicans*, *E. coli*, *S. aureus* and *S. mitis*. These microorganisms were selected for this study because, in addition to *Candida albicans* (the main etiological factor for DS), *S. aureus* and *S. mitis* have also been isolated from dentures, while *E. coli*, as part of transient microbiota, is able to promote the initial adherence of yeasts to the denture’s surface [45,46,47,48,49]. Moreover, *S. aureus* is part of ESKAPE pathogens, causative agents of the most nosocomial infections and a model for resistance [33,50]. Biofilm formation was assessed directly by the CFU count, indirectly by measuring cell viability by the MTT test, as well as being visualized by SEM. The effect of PMMA/AuNPs on decreased biofilm formation was proven by both methods. The most effective antibiofilm effect was shown for Gram-negative *E. coli*, followed by *C. albicans*, while PMMA/AuNPs were less effective against Gram-positive *S. aureus* and, especially, *S. mitis*. This effect against bacteria is in accordance with other studies, which demonstrate a strong antibacterial effect against Gram-negative bacteria and a moderate activity against Gram-positive bacteria [34]. Our SEM images also show that Gram-positive bacteria show a similar organization on PMMA with and without AuNPs. On the other hand, *E. coli* was dispersed individually on PMMA/AuNPs, while on PMMA, it showed conglomerates and initial biofilm formation. The antifungal effect of different AuNP particles [51,52] was also proved. To the best of our knowledge, this is the first study examining the antimicrobial effect of denture PMMA/AuNPs. Since AuNPs are not released from our material (indirectly proven in this study), it can be assumed that the antimicrobial mechanisms of PMMA/AuNPs are exerted through the deformation of bacterial/fungal cells. Since Gram-positive bacteria possess thicker cell walls due to the higher presence of peptidoglycans, it is not surprising that the antibacterial activity is greater on Gram-negative bacteria. Some evidence shows that AuNPs may affect the respiratory chain of aerobic respiration and lead to the generation of reactive oxygen species. Since *S. mitis* was the only bacteria grown under anaerobic conditions, this may explain why the antibiofilm activity was the lowest against *S. mitis*.

In clinical practice, since these dentures/obturators are worn for a prolonged period, it is important that the antimicrobial properties of the materials remain constant in time and that the active substance is not released. In this study, we indirectly measured the AuNP release into the medium by comparing the CFUs of microorganisms in the medium around both types of discs. Similar results of the CFUs in medium around both PMMA and PMMA/AuNP discs show that there is no antimicrobial substance released from the materials and that AuNPs are stable in the PMMA matrix.

Surface roughness (porosity) is one of the physical characteristics of materials that affects bacterial/fungal adhesion and biofilm formation [45,53,54]. Surface irregularities increase the chance of retention of microorganisms, protect them from shear forces and present micro-reservoirs of biofilms [45]. One of the factors that may affect surface roughness is residual monomer, whose vaporization increases surface roughness [55]. In our study, the amount of residual monomer was assessed indirectly by density measurements. The densities of conventional PMMA and PMMA/AuNPs were similar, implying that the surface roughness of PMMA/AuNPs is not increased due to residual monomer. Since surface roughness does not play a role in the decrease in biofilm formation on PMMA and PMMA/AuNPs, it could be assumed that the differences in their antimicrobial properties originate from AuNPs.

According to some authors, the importance of surface roughness on microbial adhesion in the mouth may be reduced compared to in vitro conditions, because of the formation and composition of salivary pellicles, which decrease surface roughness [56]. When discussing this, it should be emphasized that PMMA is mostly used for complete dentures, whose wearers are commonly elder, and obturator prothesis, mostly made after malignant tumor resections of the maxilla. Hyposalivation is frequent in elder patients, due to age and many of the medications they often use. More importantly, radiation therapy, which commonly follows tumor surgery, leads to a serious lack of saliva. Thus, in these patients, the original roughness of the material still plays a significant role in biofilm formation. In addition to the influence of density on the porosity of PMMA, a higher density of the material means a heavier denture base. It is clear that dentures should be as light as possible, which is especially important for upper dentures [19]. In this respect, the similar density of PMMA and PMMA/AuNPs could be considered as a satisfactory characteristic of the modified PMMA.

Changes in denture surface during its wear are inevitable. This is a consequence of the denture’s contact with food and cleansing procedures—toothbrushes and antiseptics. Surface hardness (wear resistance) or microhardness is a parameter that describes the resistance of materials to permanent surface indentation [57]. The wear resistance of conventional PMMA has been one of the poor mechanical characteristics of this material [58], which should be improved [59]. Through time, acquired irregularities on surfaces and increased roughness leads to impaired esthetics and higher microbial colonization on PMMA. In our study, the concentration of AuNPs slightly increased the Vicker’s microhardness. In a previous study, which prepared AuNPs using deionized water as a medium for AuNPs and which compared three different concentrations of AuNPs, the statistical increase in microhardness was detected at higher AuNP concentrations of 0.43 wt% and 0.74 wt% AuNPs [19]. Using deionized water for incorporating lyophilized AuNPs in the PMMA matrix may cause their agglomeration, which is visible as a change in the distinctive AuNP suspension color from red to purple or blue. The use of the USP production method and lyophilization offered the advantage of producing dried AuNPs, which were able to be redissolved in suspensions in different media, such as ethanol. Other advantages of this production method are the simplicity of setup and adaptability, and the continuous operation and cost-effectiveness of USP, with good possibilities for production scale up. Through experimental work, the AuNPs were visibly more stable in ethanol as a solvent, and this was the reason why we decided to synthetize PMMA with AuNPs dissolved in ethanol. However, this mechanical characteristic should be improved in further investigations. As the mechanical characteristics of the composite are comparable to the conventional PMMA, the possibility of use of these composites is also considered comparable to the conventional PMMA, widely used in dentistry.

The addition of nanoparticles that increase the antimicrobial properties is desirable in materials for denture base/obturators, since the conventional PMMA does not possesses this characteristic. Denture biofilms present a dense microbial community comprising up to 10^11^ microorganisms per milligram [60]. As previously mentioned, dentures are reservoirs of these microorganisms, and they may detach from the denture and disseminate. The aspiration of oropharyngeal contents commonly occurs in healthy subjects, while about 45% aspirate into the lungs during sleep [14,61]. Additionally, these microorganisms grown in a biofilm may be up to 1000 times more resistant to antibiotics/antifungals than planktonic cells [62,63]. On the other hand, although denture stomatitis is a benign and mostly asymptomatic inflammation of the oral palatal mucosa, DS therapy has shown rapid recurrence probably as consequence of recontamination by yeasts [64] and a lack of dexterity of the wearers of dentures/obturators. This implies additional antifungal therapy, with the previously mentioned side effects. Since the oral cavity is a nutrient-rich environment, which favors biofilm formation, and the surface of the denture, which is in contact with the palatal mucosa, is non-polished and hidden from self-cleaning by mastication, the importance of preventing biofilm formation is essential.

Even though the modification of PMMA with AuNPs increases the cost of denture/obturator fabrication, AuNPs in an ethanol solution are stable for a longer period and the procedure of PMMA/AuNP denture production is not more complicated than conventional dentures, which leads to only a slightly increased price of dentures modified by AuNPs. This increase in denture price may be compared to the price of therapy of repeated DS and its potential complications. Nevertheless, the fact that the patients do not develop complications associated with dentures’ biofilm would increase the patient’s satisfaction with the improved product.

## 5. Conclusions

It can be concluded that PMMA/AuNPs showed a significant antibiofilm effect against the monomicrobial biofilm formation of *C. albicans*, *S. mitis*, *S. aureus* and *E. coli*. This effect is the strongest against Gram-negative *bacteria* and *C. albicans* than it is against Gram-positive bacteria. Density is similar between PMMA and PMMA/AuNPs. Microhardness is slightly increased in PMMA/AuNPs compared to that in the conventional PMMA.

## Figures and Tables

**Table 1:** Growth conditions of the reference strains used for monomicrobial biofilm formation.

Reference Strain	Growth Medium	Temperature	Time	Conditions
*Candida albicans*	Saburo Agar *	37 °C	24 h	Aerobic
*Echerichia coli*	Endo Agar *	37 °C	24 h	Aerobic
*Staphylococcus aureus*	Columbia agar with 5% sheep blood **	37 °C	24 h	Aerobic
*Streptococcus mitis*		37 °C	48 h	Anaerobic

Manufacturer of the growth medium: * HIMEDIA (Mumbai, India); ** ProReady (Kikinda, Serbia).

**Table 2:** The results of the Vickers microhardness for the PMMA and PMMA/AuNP laminae.

	Mean ± SD	Minimum	Maximum
PMMA (MPa)	18.59 ± 1.39	17.07	20.62
PMMA/AuNPs (MPa)	19.18 ± 1.18	17.05	21.52

**Table 3:** Results of the density measurements for the PMMA and PMMA/AuNP laminae.

	Mean ± SD	Minimum	Maximum
PMMA (g/cm^3^)	1.17 ± 0.01	1.16	1.17
PMMA/AuNPs	1.17 ± 0.03	1.14	1.23

## Data Availability

The data presented in this study are available on request from the corresponding author. The data are not publicly available due to these results are part of ongoing doctorial dissertation.
